# Comparing silver and gold nanoislands’ surface plasmon resonance for bisacodyl and its metabolite quantification in human plasma

**DOI:** 10.1186/s13065-024-01157-8

**Published:** 2024-03-23

**Authors:** Khadiga M. Kelani, Maha M. Ibrahim, Nesreen K. Ramadan, Eman S. Elzanfaly, Sherif M. Eid

**Affiliations:** 1 Analytical Chemistry Department, Faculty of Pharmacy, Cairo University, Cairo, Egypt; 2 Analytical Chemistry Department, Faculty of Pharmacy, Modern University for Technology and Information, Cairo, Egypt; 3 Pharmaceutical Chemistry Department, Faculty of Pharmacy and Drug Technology, Egyptian Chinese University, Cairo, Egypt; 4 Analytical Chemistry Department, Faculty of Pharmacy, 6 October University, October City, Egypt

**Keywords:** Optical biosensor, Localized surface plasmon resonance, Surface-enhanced infrared absorption spectroscopy, Partial least squares, Bisacodyl, Plasma

## Abstract

Gold and silver nanoparticles have witnessed increased scientific interest due to their colourful colloidal solutions and exceptional applications. Comparing the localized surface plasmon resonance (LSPR) of gold and silver nanoparticles is crucial for understanding and optimizing their optical properties. This comparison informs the design of highly sensitive plasmonic sensors, aids in selecting the most suitable nanoparticles for applications like surface-enhanced infrared spectroscopy (SEIRA) and biomedical imaging, and guides the choice between gold and silver nanoparticles based on their catalytic and photothermal properties. Ultimately, the study of LSPR facilitates the tailored use of these nanoparticles in diverse scientific and technological applications. Two SEIRA methods combined with partial least squares regression (PLSR) chemometric tools were developed. This development is based on the synthesis of homogeneous, high-dense deposited metal nanoparticle islands over the surface of glass substrates to be used as lab-on-chip SEIRA sensors for the determination of bisacodyl (BIS) and its active metabolite in plasma. SEM micrographs revealed the formation of metallic islands of colloidal citrate-capped gold and silver nanoparticles of average sizes of 29.7 and 15 nm, respectively. BIS and its active metabolite were placed on the nanoparticles’ coated substrates to be directly measured, then PLSR chemometric modelling was used for the quantitative determinations. Plasmonic citrate-capped gold nanoparticle substrates showed better performance than those prepared using citrate-capped silver nanoparticles in terms of preparation time, enhancement factor, PLSR model prediction, and quantitative results. This study offers a way to determine BIS and its active metabolite in the concentration range 15–240 ng/mL in human plasma using inexpensive disposable glass-coated substrates that can be prepared in 1 h to get results in seconds with good recovery between 98.77 and 100.64%. The sensors provided fast, simple, selective, molecular-specific and inexpensive procedures to determine molecules in their pure form and biological fluid.

**Supplementary Information:**

The online version contains supplementary material available at 10.1186/s13065-024-01157-8.

## Introduction

Gold and silver nanoparticles show unique physical and chemical characteristics and bioactivity making them the widely used nanoparticles in bioanalysis [1], bioimaging [2], antibacterial [3], nano-sensing [4], dairy products analysis [5] and other nanotechnology applications [6]. These metal nanoparticles showed characteristic well-defined and distinct plasmon absorption in visible light known as localized surface plasmon resonance (LSPR) [7]. LSPR phenomena cause enhancement of the absorption bands and/or increased scattering intensity at certain light wavelengths absorbed by gold or silver nanoparticles [7, 8]. LSPR of the gold or silver nanoparticles is related to the nanoparticle shape, size, interparticle distance, composition, and refractive index (dielectric constant) of the surrounding medium [4, 7, 9–14]. This LSPR phenomenon was the driving force for the development of different advanced techniques such as surface-enhanced Raman spectroscopy (SERS) [15, 16], surface-enhanced infrared spectroscopy (SEIRA) [17–21], and tip-enhanced infrared nano-spectroscopy (TEIRA) [22].

Several studies have been devoted to answering the question of, which is better gold or silver nanoparticles, and the comparison of their preparation and characteristics [7, 11, 14, 15]. A study devoted to studying the softness of silver in comparison to gold nanoparticles examined the metal-ligand interaction of the nanoparticles employing nitrogen and sulphur as soft and borderline donor atoms [14]. Another study compared the preparation process and stability of silver and gold nanoparticles [11]. Other researchers compared the negatively charged silver and gold nanoparticles using hydrophilic thiols as SERS probes for structural similarities and differences [15].

In this study, we have compared the performance of silver and gold nanoparticles as SEIRA probes for the analysis of a pharmaceutical active ingredient (Bisacodyl acetate) and its biological metabolite. We have explored the preparation process of such nanomaterials, details on their chemical and physical characterization, stability, interaction with the under-investigation molecules, enhancement factors, and applicability of SEIRA on biological samples.

Surface-enhanced infrared absorption (SEIRA) [17–21] is considered an advanced Fourier transform infrared spectroscopy (FTIR) analysis [23, 24] that combines the power of vibrational spectroscopy with nanotechnology and chemometrics [25, 26]. Several applications have been described for SEIRA [10, 13, 19–21]. In SEIRA, the enhancement of the infrared (IR) signal is highly dependent on the electric field enhancement because of LSPR resulting from the thin metal islands of silver or gold nanoparticles on the substrate surface.

The coupling between SEIRA and chemometric tools such as partial least squares regression (PLSR) [25–27] opened the way for different applications, due to the ability of PLSR to explain and interpret the highly complicated SEIRA bands providing qualitative and quantitative data that can be used for different applications. PLSR can make quantitative predictions even in the presence of interfering compounds using the whole SEIRA spectrum without ignoring any part of the spectrum that may include important information. Several methods have applied PLSR chemometric calculation for the analysis of pharmaceutical components [21, 27, 28].

The coupling between SEIRA and PLSR has been applied for simultaneous analysis of Bisacodyl acetate (BIS) and its metabolite, that used as a model for comparing silver and gold nanoparticles LSPR. Bisacodyl acetate is a derivative of pyridinyl methylene diacetate ester which acts as a laxative indicated to treat bowel irregularity and constipation and to clean out the intestines before a bowel examination and surgery [29, 30]. Bis-(p-hydroxyphenyl)-pyridyl-2-methane is the deacylated active form of BIS that is developed due to intestinal metabolism by deacetylase and bacterial enzymes which is responsible for the laxative action [31, 32]. Quantitative determination of BIS and its metabolite in biological samples is of specific importance as there are several reports of its laxative poisoning at certain doses [33], BIS can increase estrogen activity in breast cancer cells [34] and its cytotoxicity on human stem cells [35].

Various analytical methods were reported for quantitative analysis of BIS and its active metabolite including capillary electrophoresis [36], liquid and gas chromatography [37–39], thin layer chromatography [40], spectrophotometry [41, 42] and electrochemical methods [43, 44]. SEIRA could provide many advantages over the chromatographic methods due to portability, simplicity, short operation time, not requiring skilled operator, fewer trials, minimal waste generated, less sample size, and it is not affected by changes in temperature. In addition, SEIRA can be used for the analysis of samples as solid, liquid, or gas without tedious treatment. Also, the IR spectrum is considered a fingerprint for each molecule.

**Fig. 1:**
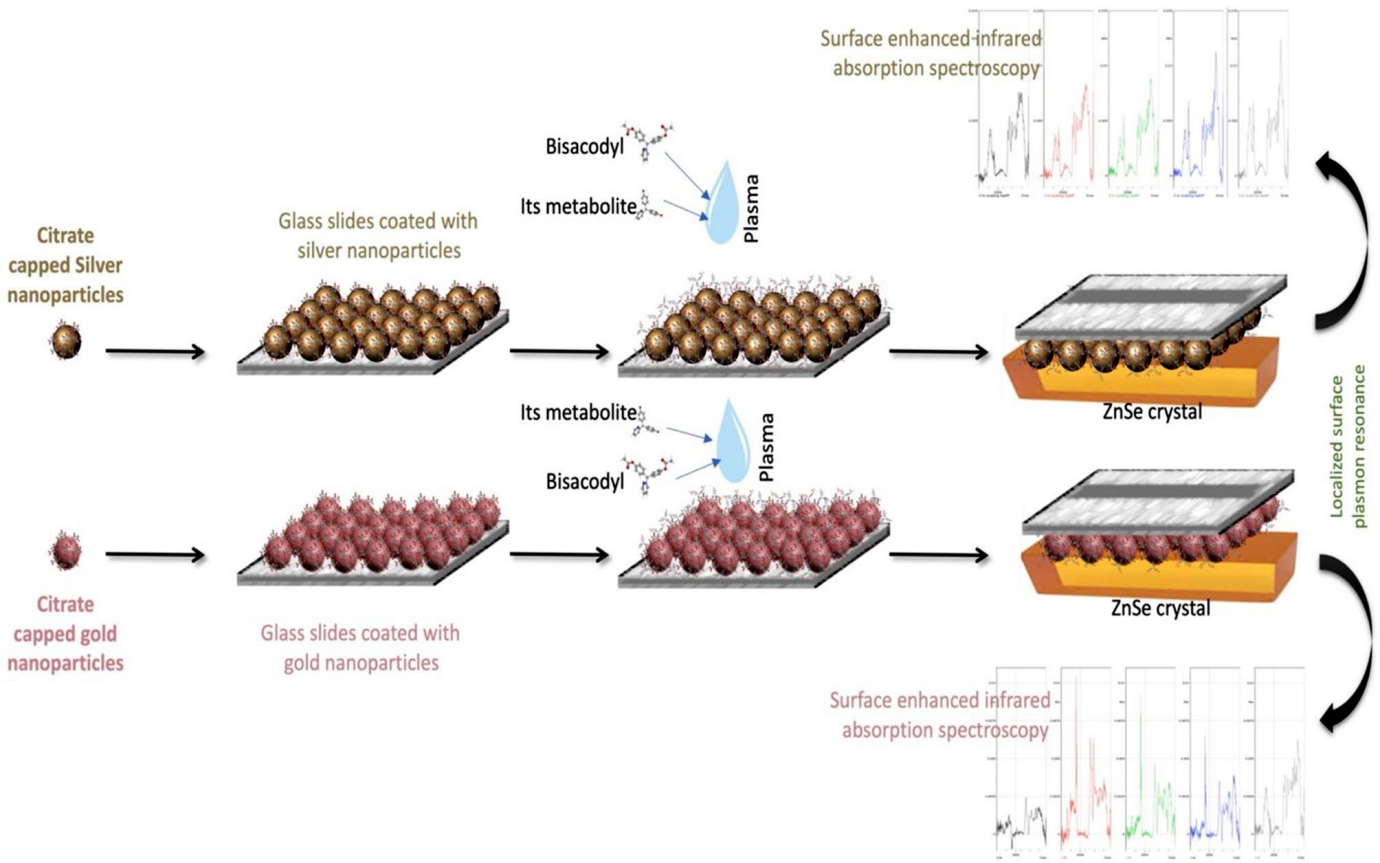
Overview of the simultaneous determination of BIS and its metabolite in human plasma using the SEIRA-PLS method

The preparation of current SEIRA sensors included a one-step synthesis of negatively charged citrate-coated gold nanoparticles (Cit-AuNPs) and citrate-coated silver nanoparticles (Cit-AgNPs) followed by a coating step of treated glass substrates with these nanoparticles as illustrated in Fig. 1. Several characterization steps using UV/VIS, FTIR, ZetaSizer, transmission electron microscope (TEM), and Scanning electron microscope (SEM) were performed to confirm the proper preparation and deposition. Then, the performance of Cit-AuNPs and Cit-AgNPs coated substrates was evaluated for the quantitative determination of pharmaceuticals in biological fluids using BIS and its metabolite as model drugs for the analysis.

## Experimental details

### Software and instruments

An IR Affinity-1 FTIR instrument, Shimadzu Japan, was utilized for data collection equipped with a ceramic light source, a temperature controller, a Ge-coated KBr as a beam splitter, and a Deuterated Lanthanum α Alanine doped TriGlycine Sulphate detector. A horizontal attenuated total reflectance (ATR) unit was connected to the FTIR instrument. The ATR unit is equipped with a trapezoid-shaped Zinc Selenide (ZnSe) crystal 8 cm long, 1 cm wide, and 0.4 cm depth. The IR Affinity-1 FTIR instrument operated with IR solution software that was adjusted to the following parameters: Measurement mode: Absorbance, Apodization Function: SqrTriangle, Mid-IR scanning range: 400–4600 cm^− 1^, Resolution: 4 cm^− 1^, Number of scans: 50, Mirror speed: 2.8. Unscrambler X® version 10.4 software used for PLSR modelling using the experimental set concentrations as the predictors set and SEIRA data points as the responses set. The method was adjusted to use random cross-validation at the optimum number of components. Double beam Shimadzu spectrophotometer type 1800-PC Japan equipped with a 1 cm quartz cuvette and controlled using UV Probe V.2.4 software adjusted to a wavelength between 800 and 200 cm^− 1^, scanning speed: fast, and sampling interval of 0.1. The nanoparticle’s hydrodynamic radius and Zeta potential were calculated based on dynamic light scattering calculations using the ZetaSizer device model Nano ZS90, Japan. The device is equipped with Helium–Neon laser light, a wavelength of 633 nm, and a backscattered detector at an angle of 173°. The device is operated by Malvern software V.7.11 (Malvern, Japan). Transmission electron microscope micrographs (TEM) were recorded by a JOEL JEM-1010 (Japan) at an acceleration voltage of 80 kV. The micrographs of the scanning electron microscope (SEM) were examined using a Quanta 250 FEG (UK) at 30 kV quickening voltage.

### Materials

Standard BIS (purity 99.24%), standards of trisodium citrate (TC), silver nitrate (AgNO_3_), gold chloride trihydrate, 3-aminopropyl triethoxysilane (APTES), and methanol with purity 99% were purchased from (Sigma-Aldrich, USA). Human plasma 500 mL was obtained from a blood bank, VACSERA, Giza, Egypt. They informed that it was collected from white male healthy volunteers aged between 29 and 30 years old. Clean glass slides of 1.2 mm thickness and 25.4 × 76.2 mm diameter were obtained from (Sigma-Aldrich, USA). Sodium hydroxide (NaOH) and hydrochloric acid (HCl) were obtained from Biochem Company, Egypt. A Milli-Q device (Millipore, USA) was used to prepare high-purity deionized water.

### Solutions

#### Preparation and isolation of bisacodyl active metabolite

The active metabolite of BIS was prepared by chemical degradation of BIS followed by chromatographic separation of the metabolite. In a stoppered flask, accurately weighed 1 gm of BIS was transferred and dissolved into 200 mL of 0.10 M HCl, then left for 24 h at room temperature. The solution was neutralized using 1.00 M NaOH to obtain a pH of 6.00, then filtered to get a precipitate consisting of two degradation products Monoacetyl BIS and Desacetyl BIS as illustrated in Fig. 1S [45]. Desacetyl BIS is the active metabolite of BIS [46] that we have separated following the steps described by Metwally et al. [47]. A preparative normal phase column chromatography has been performed for separation of the active metabolite (Desacetyl BIS) from other degradation products using a mobile phase of acetone: chloroform (1:9 v/v). The complete separation was confirmed by several TLC sampling and FTIR scanning as the FTIR spectrum considered as a fingerprint of the compound [27, 48].

#### Stock and working solutions

Exact 0.01gm of BIS and its active metabolite were accurately weighed and transferred into two 10-mL flasks separately. The volumes were completed using methanol to get a standard stock solution of (1 mg/mL). Then the working solutions (15–240 ng/mL) for both BIS and its active metabolite were prepared by diluting the stock solution appropriately.

#### Preparation of Cit-AgNPs

According to the Lee-Meisel technique [49], the stabilized Cit-AgNPs were synthesized by the reduction of AgNO_3_ with TCA. Exactly 36.00 mg of powdered AgNO_3_ were placed in a glass beaker and dissolved in 350 mL of double distilled water. The solution was continuously stirred and heated to 70 °C then 5 mL of freshly prepared TCA (5%) was added dropwise. The appearance of yellow color indicated the formation of Cit-AgNPs. The solution was centrifugated for 10 min at 6000 rpm to remove the unreacted TCA and to get the Cit-AgNPs precipitated. Centrifugation was repeated several times to ensure complete precipitation. The precipitated nanoparticles were collected, then carefully redistributed in water and stored at 4 °C.

#### Preparation of Cit-AuNPs

The reduction of gold (III) chloride trihydrate by sodium citrate in water is an easy technique for AuNPs synthesis, which was first described by Turkevich in 1951 [50]. Exactly 36.00 mg of gold chloride trihydrate were transferred into a glass container and dissolved in 350 mL of double distilled water accompanied by continuous shaking and heating to 70 °C. Then TCA solution (5%) was added dropwise. After about 20 min, the light-yellow solution was changed to wine red, indicating the formation of Cit-AuNPs. The solution was centrifugated for 10 min at 6000 rpm to remove the unreacted TCA and to get the Cit-AuNPs precipitated. Centrifugation was repeated several times to ensure complete precipitation. The precipitated nanoparticles were collected, then carefully redistributed in water, and stored at 4 °C.

### Coating of glass slides with Cit-AgNPs or Cit-AuNPs

Clean glass slides were cut to 1.00 cm in length and 0.50 cm in width and immersed in a mixture of methanol and concentrated HCl (1:1 v/v) for 35 min to degrease the surface of the glass slides. The slides were removed from the methanolic solution and washed several times with pure water to remove excess HCl to prevent its interaction with reagents in the next steps. Further cleaning of the glass surface was performed by immersing the slides in piranha solution (consisting of H_2_SO_4_: H_2_O_2_ (3:1 v/v)) for one hour to reveal the oxygen groups of the surfaces of the glass slides then carefully washed with pure water multiple times. The cleaned glass slides were then salinized by immersing in 1% APTES for two hours to get a salinized glass. Finally, the slides were washed several times using absolute ethanol to clean the slides from excess APTES and heated in the oven for one hour at 120ºC.

The coating of the salinized glass slides with the prepared nanoparticles was performed using the wet chemical deposition method [9, 17, 51]. This method is based on the soaking of salinized glass slides in solutions of nanoparticles for different durations called different soaking times to get the nanoparticles deposited on the surface of the glass. The salinized glass slides were placed into the freshly prepared Cit-AgNPs or Cit-AuNPs solutions at different intervals of soaking time (1, 2, 3, and 4 h). Then the glass slides were placed into an oven adjusted to 100 °C for 10 min to enhance the binding of the nanoparticles to APTES on the glass slides’ surface. Then SEM micrographs were recorded.

### Measurements of FTIR spectra

The ATR unit was first connected to the FTIR device. To avoid the interference of moisture or carbon dioxide during the measurements, the device was purged with a dry nitrogen gas. For each sample measurement, about 500 µL of the sample mixture (BIS and its active metabolite) was dropped onto the coated glass slide and left for complete dryness. The dried slides were then placed over the ZnSe prism of the ATR unit to get the sample in direct contact with the surface of the ZnSe prism, and then the FTIR scanning was started. The IRsolution software was adjusted to the following parameters: IR region 4500–400 cm^− 1^, scan number 50, resolution 4, and apodization function is Sqr-triangle. Each measurement provides an FTIR spectrum of 2100 data points utilizing a wavenumber interval of 1.95 and is stored on a computer.

The infrared light passes through the horizontal ZnSe prism at an angle of 45° and it reflects about 14 times, producing an evanescent light that enters around 2 mm inside the under-investigation sample. The calculated refractive index of the ZnSe prism was observed to be 2.43 and the light path length was equal to 12.13 mm.

### Calibration and validation of SEIRA-PLSR chemometric model

The multilevel partial factorial design [26, 52] was applied for the design of 25 mixtures as a calibration set using mixtures of BIS and its active metabolite dissolved in methanol/plasma as a solvent. The calibration set was in the concentration range of 15–240 ng/mL for both BIS and its metabolite (Table 1S). For each measurement, 500 µL from each mixture was dropped over the coated glass slide and left for complete dryness. Then, the dried slides were placed over the ZnSe prism of the ATR unit to get the sample in direct contact with the surface of the ZnSe prism, and then the FTIR scanning was started. Each of the mixtures was scanned 45 times and averaged. Other 10 mixtures were prepared and considered as the validation set.

### Application of the method to spiked human plasma

Accurately measured 1.00 mL of the sample (mixture of BIS and metabolite) dissolved in methanol was injected into 1.00 mL of plasma and 1.00 mL of methanol so that the volume of methanol was twice the volume of plasma for protein precipitation [29, 30]. The mixtures were vortexed for 5 min followed by centrifugation at 5000 rpm for 10 min. Plasma samples were spiked using different concentrations of BIS and its active metabolite considering that the selected concentrations were close to the reported values (26 and 236 ng/mL) of BIS C_max_ for tablets and oral solution, respectively [53]. The determination of BIS and the active metabolite in human plasma was achieved by using the developed PLSR-SEIRA method by placing 500 µL on the surface of glass slides coated with Cit-AuNP.

## Results and discussion

The developed method demonstrated the performance of Cit-AgNPs and Cit-AuNPs coated glass slides for the rapid quantitative determination of structurally related compounds as BIS and its active metabolite (disacetyl bisacodyl).

### Nanoparticles’ characterization

**Fig. 2:**
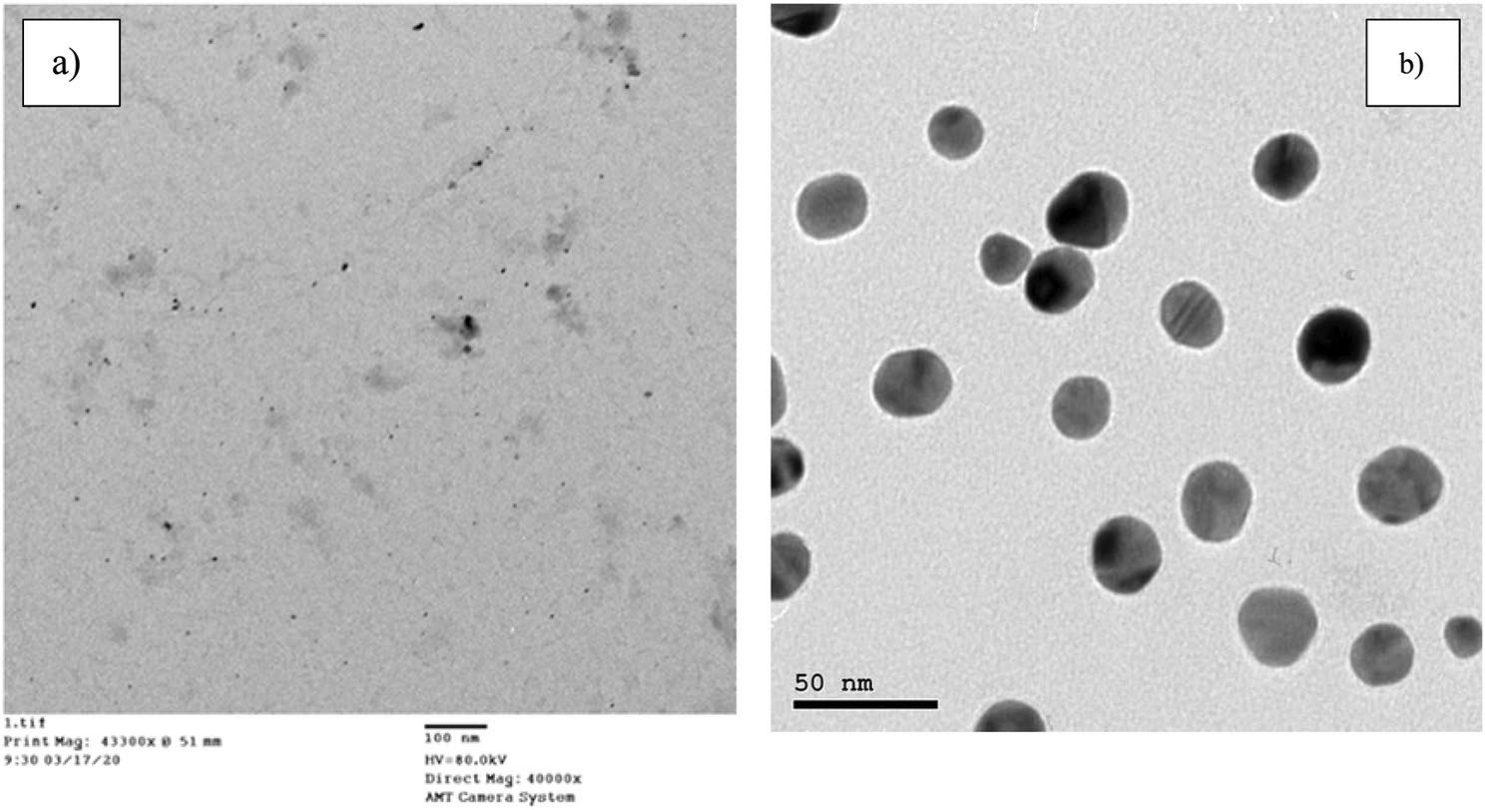
TEM micrographs of **(a)** Cit-AgNPs and **(b)** Cit-AuNPs

The prepared Cit-AgNPs and Cit-AuNPs showed a characteristic UV/VIS spectrum as presented in Fig. (2S). The characteristic UV/VIS spectra of the Cit-AgNPs and Cit-AuNPs nanoparticles appeared as narrow peaks at λ_max_ of 415 and 525 nm, respectively, which indicated that the prepared nanoparticles were spherical or mainly spherical and their size between 10 and 60 nm as previously reported [54–56].

**Fig. 3:**
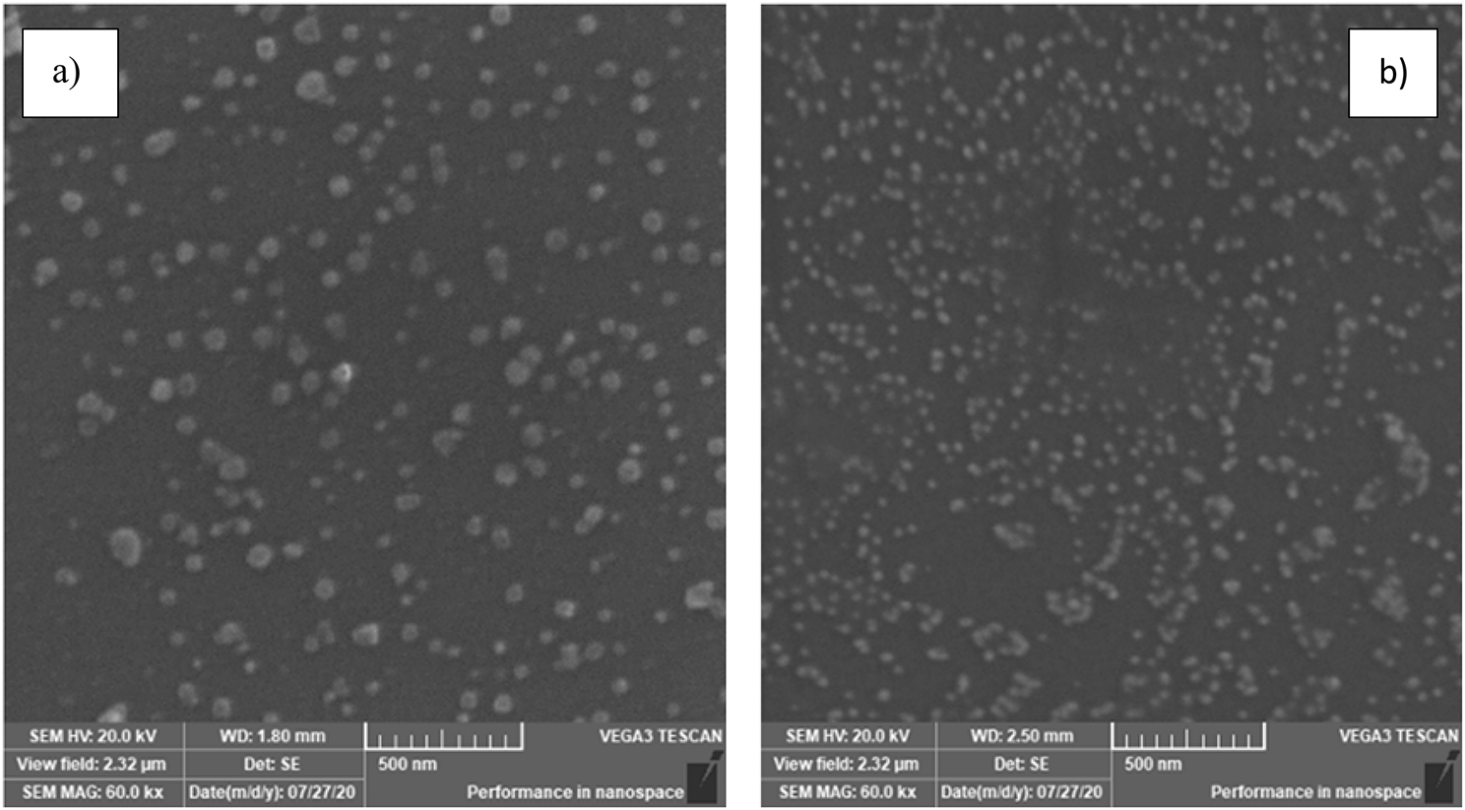
SEM micrographs of glass slides coated by **(a)** Cit-AgNPs (soaking time:4 h) and **(b)** Cit-AuNPs (soaking time:1 h)

The prepared Cit-AgNPs and Cit-AuNPs were analyzed three times by Zeta-sizer at 25 °C with an angle of incidence equal to 90° using dynamic light scattering calculations. The average hydrodynamic radius for both Cit-AgNPs and Cit-AuNPs were 15 and 29.7 nm, respectively as shown in Fig. 3S. The polydispersity index (PDI) obtained was 0.641 and 0.457 respectively, which were in the range of the PDI reference values of 0.1–0.7, indicating monodispersed nanoparticles [57].

**Fig. 4:**
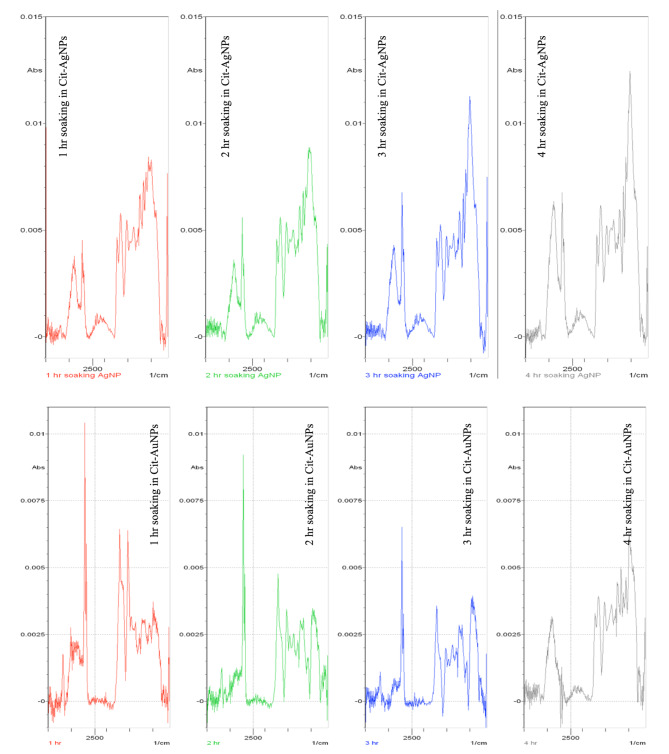
SEIRA spectra of the mixture number 21 of equal concentrations (120 ng/mL) of BIS and its active metabolite on the surface of AgNPs and nanoparticle-coated coated glass slides. The same concentration of the mixture was measured on the surface of glass slides soaked for 1, 2, 3, and 4 h in solutions of Cit-AgNPs and Cit-AuNPs

Two experiments were performed to ensure the stability of the prepared Cit-AgNPs and Cit-AuNPs. The first experiment was performed by recording the UV/VIS absorption spectra of Cit-AgNPs and Cit-AuNPs after a month of preparation, and the stability duration of the nanoparticles ensured based on; the shape, width, and λ_max_ of the UV/VIS spectra that remained unchanged during the measurement period. The second experiment was the measurement of the Zeta potential [58–60] of nanoparticles at different time intervals, focussing on recording the charge on the outer surface of the prepared Cit-AgNPs and Cit-AuNPs. Figure 4S shows the results of the stability experiments, Fig. (4S-A) Plot of Zeta potential measured during 30 days for Cit-AuNPs and Cit-AgNPs indicating negatively charged nanoparticles for the whole duration of the 30 days allowing electrostatic repulsion preventing agglomeration. Figure 4S-B & C) shows the UV/Vis spectrum of Cit-AuNPs measured at the beginning and the end of the 30 days indicating that the peak shape, width, and λ_max_ remained unchanged during the measurement period. Figure (4S-C & D) shows the UV/Vis spectrum of Cit-AgNPs measured at the beginning and the end of the 30 days indicating that the peak shape, width, and λ_max_ remained unchanged during the measurement period. The average values of zeta-potentials were found to be -30 and − 29 mV for Cit-AgNPs and Cit-AuNPs, respectively, indicating strong negatively charged nanoparticles. This negative charge onto the nanoparticles’ surface allows electrostatic repulsion that prevents agglomeration and increases stabilization of the formed nanoparticles [58, 59].

TEM micrographs used for identifying the size, shape, and dimensions of the prepared nanoparticle. A drop of the prepared nanoparticles was placed on the surface of a carbon-coated copper grid. After the complete drying of the grid, it was placed inside the measurement holder to be scanned using the TEM device. TEM micrographs (Fig. 2) indicated uniform spherical or semi-spherical Cit-AgNPs and Cit-AuNPs. They indicated that the sizes of the nanoparticles were in the range of 10 to 60 nm which is in agreement with the results of ZetaSizer that the hydrodynamic radius of gold nanoparticles is 29.7 nm, and the hydrodynamic radius of silver nanoparticles is 15 nm.

SEM micrographs were used for scanning the shape, size, and morphology of the nanoparticle metal islands formed on the surface of the glass slides. The nanoparticle-coated glass slides were placed in the SEM device. Figure 3 showed SEM micrographs indicating that the deposited Cit-AgNPs and Cit-AuNPs created metal islands that absorb the infrared light, leading to the LSPR magnification of the surrounding molecules. A smooth surface was observed with no irregular shapes. It tends to be seen from Fig. 4 that the highest enhancement in SEIRA peaks occurs after 4 h and 1 h for slides coated with Cit-AgNPs or Cit-AuNPs, respectively. This is in accordance with the obtained SEM micrographs for the slides soaked in silver and gold nanoparticles, respectively. In the case of gold-coated nanoparticles, SEM micrographs of the soaked slide for 1 h showed the best uniformity of the gold nanoparticles islands as shown in Fig. 3b, while the soaked slides for 2,3 and 4 h showed aggregates and less uniformity; this comes in accordance with the SEIRA measurements as shown in Fig. 4, the enhancement after 1 h was the best while the other times showed irregular / less SEIRA enhancement. In case of silver-coated nanoparticles, SEM micrographs of the soaked slides for 4 h showed the best uniformity of silver nanoparticles islands as shown in Fig. 3a, while the slides that have been soaked for less than 4 h showed less uniformity of the nanoparticles’ islands in their SEM micrographs. This led to lower SEIRA enhancement for the slides soaked 1, 2 and 3 h and a higher enhancement for the slides soaked for 4 h as shown in Fig. 4. upon soaking of the slides into silver nanoparticles for longer durations (5 and 6 h), the nanoparticles showed aggregation that led to irregular weak SEIRA enhancement confirming that soaking the slides for 4 h was the best.

### The effect of LSPR of the nanoparticles on the bands of the molecules

**Fig. 5:**
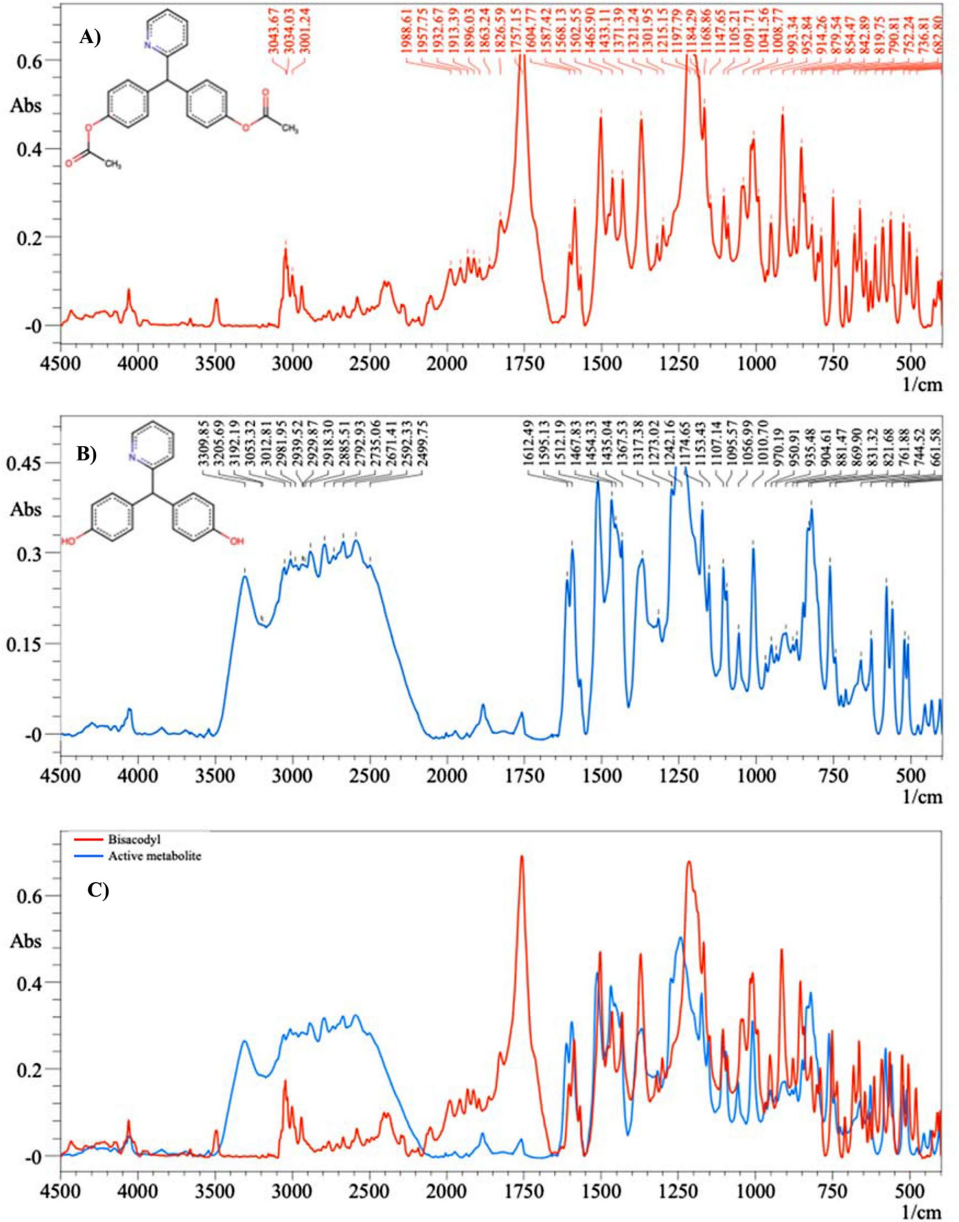
The molecular structures and the SEIRA absorption spectra on Cit-AuNPs of **(A)** 0.2 µg/mL Bisacodyl, **(B)** Active metabolite 0.2 µg/mL and **(C)** the overlay plot of both compounds

The infrared bands were significantly enhanced when an infrared active substance was near Cit-AgNPs or Cit-AuNPs; this phenomenon is known as LSPR enhancement (SEIRA due to LSPR). Mixtures of BIS and its active metabolite were used as an infrared probe molecule to investigate the SEIRA activity of the prepared gold and silver nanoparticles. Figure 5 presents the SEIRA extension spectra of BIS and its active metabolite absorbed on the surface of negatively charged Cit-AuNP. Table 1 illustrates a comparison of the obtained enhanced IR peaks and the infrared peaks reported in the literature on BIS and its active metabolites. It represents the shifts of the main peaks and the fingerprint ranges of the drug, and its active metabolite accompanied by their assignments. Different SEIRA-absorbed bands for BIS and its metabolite were observed in the selected infrared range. The peaks at 1755 cm^− 1^ showed the stretching vibration of the C = O bond, which disappeared in the FTIR spectrum of its metabolite. Furthermore, the appearance of O-H stretching in the range of 3500 − 3100 cm^− 1^ indicated the formation of O-H groups in the structure of the metabolite.

**Fig. 6:**
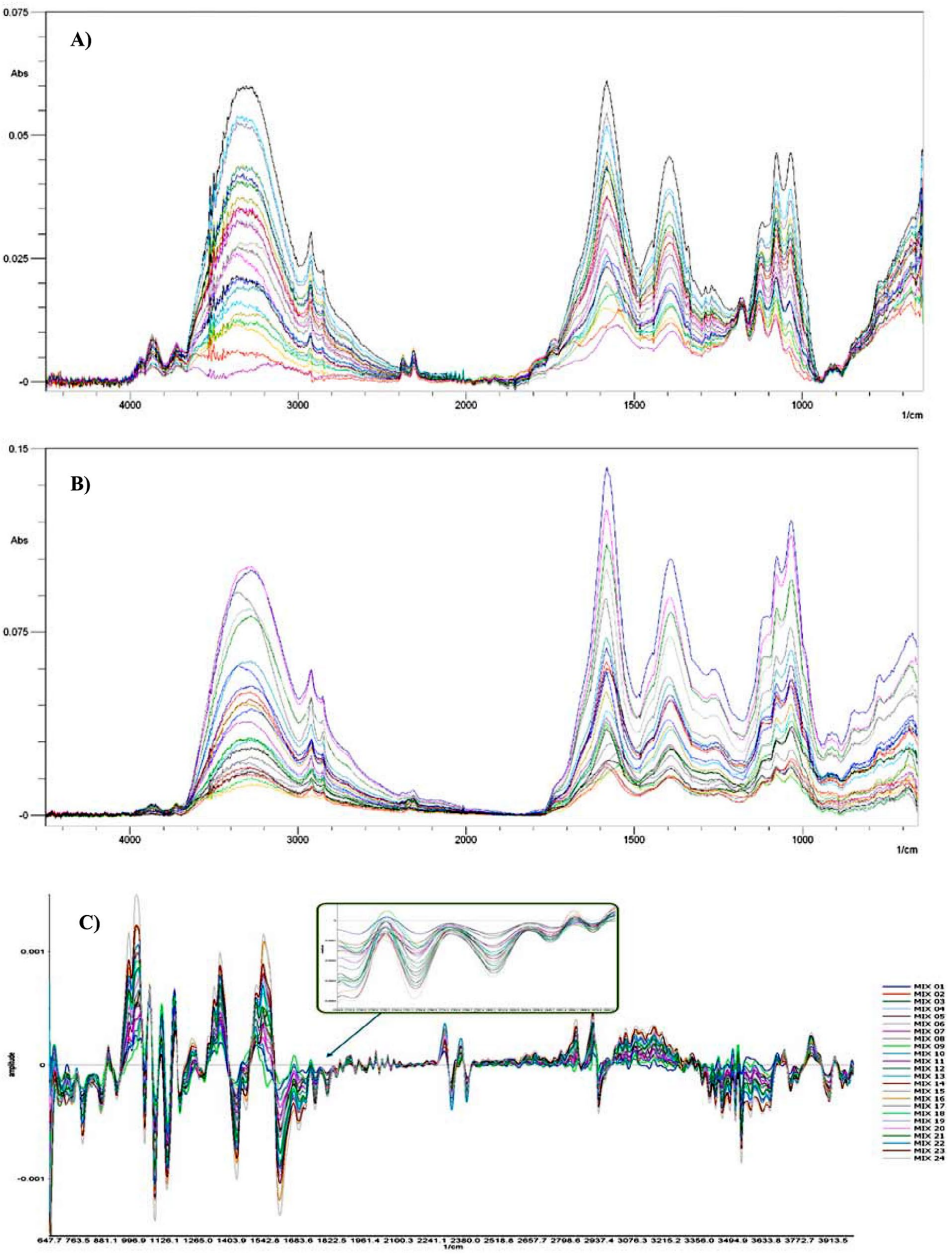
The overlay construction of the 25 mixtures of BIS and its active metabolite measured on the surface of a glass substrate coated with **(A)** Cit-AgNPs, **(B)** Cit-AuNPs, and **(C)** Savitzky-Golay first derivative algorithm of mixtures adsorbed on Cit-AuNPs

Upon comparing the SEIRA spectra of the mixtures of BIS and its metabolite obtained on the surface of AgNPs-coated glass slides to that obtained on the surface of AuNPs-coated glass slides, it was observed that the positions of the bands remained unchanged. However, notable difference was observed solely in the intensities of the bands, as shown in Fig. 6A and B. This may be due to that both gold and silver nanoparticles exhibit surface plasmon resonance leading to local electromagnetic field enhancements around the nanoparticles. However, the degree of enhancement can vary between gold and silver due to differences in their plasmonic properties. This difference can affect the intensity of the SEIRA signals. Also, the absorption cross-section, which relates to how efficiently a molecule absorbs infrared radiation, can be influenced by the local electromagnetic field enhancement. Differences in the absorption cross-section between gold and silver can lead to variations in peak intensities. Moreover, the resonance conditions for gold and silver nanoparticles may not be identical, leading to variations in the efficiency of energy transfer between the nanoparticle and the adsorbed molecules. This can affect the overall intensity of the SEIRA signals.

**Table 1:** Infrared absorption bands and different modes of vibration for BIS and its active metabolite

Studied Drug	Groups	Peak assignment	Expected and/or reported values peak (cm^− 1^)	Present method values peak (cm^− 1^)
***Biscodyl***	C-O of the ester group	C-O	1250–1310	1301
	The tertiary amine of the pyridine ring	C = N	1640–1690	1640
	Carbonyl	C = O stretching	1690–1760	1755
***Active*** ***Metabolite***	Aromatic alcohol	O-H	3200–3650	3309
	The tertiary amine of the pyridine ring	C = N	1640–1690	1640
	Alcohol	C-OH	1110–1337	1273

The surface of the glass substrate with and without the nanoparticles was used to examine the magnitude of amplification for the infrared band. The SEIRA spectra of BIS and its metabolite mixture placed on glass slides coated with Cit-AgNPs (prepared by different soaking times) were measured. The suitable background spectrum of glass slides coated by Cit-AgNPs (prepared by different soaking times) was used for each measurement. The same measurements were performed using Cit-AuNPs (prepared by different soaking times) as presented in Fig. 4. It was found that the recorded SEIRA band intensities increase till four hours of soaking for Cit-AgNPs and one hour for Cit-AuNPs, and then irregular bands appear at higher soaking durations. These results came in accordance with the SEM micrographs presented in Fig. 3 that indicated the uniform semi-spherical/ spherical Cit-AgNPs or Cit-AuNPs on the glass substrate surfaces that were soaked for the same time intervals.

The enhancement factor (EF) was calculated using the following Eqs. [16, 61, 62]:-

$$EF= \frac{{I}_{SEIRA} / {C}_{SEIRA}}{{I}_{Normal} / {C}_{Normal}}$$

Where *EF* is the enhancement factor, ${I}_{SEIRA}$ and ${I}_{Normal}$ are the intensities of the same band for SEIRA and the normal FTIR spectra. ${C}_{SEIRA}$ and ${C}_{Normal}$ are the concentrations for the samples used for SEIRA and the normal FTIR spectra. The coating of the glass substrates using Cit-AgNPs caused about two-fold enhancement of the SEIRA signal of BIS and its active metabolite signal while Cit-AuNPs cause about three-fold enhancement of the same mixture SEIRA signals. Similar observation was reported by Martínez-Hernández et al. (2021) on Hg^2+^ detection based on the LSPR of silver and gold nanoparticles using an optical fiber sensor. The LSPR-AuNPs were much more sensitive to the presence of Hg^2+^ than LSPR-AgNPs [63].

Each image in Fig. 4 related to glass slides soaked in the nanoparticles for different duration (1, 2, 3, and 4 h), During soaking, the nanoparticles bind to the surface of the glass forming metal islands. These islands differ in size, shape, uniformity, and agglomeration state leading to differences in localized surface plasmon resonance of the analytes measured on its surface. In the case of gold nanoparticles, the best nanoislands formed after 1 h of soaking as shown in Fig. 3b, leading to the best enhancement as shown in Fig. 4. In the case of silver nanoparticles, the best nanoislands formed after 4 h of soaking as shown in Fig. 3a, leading to the best enhancement as shown in Fig. 4. The proposed mechanisms to elucidate the interaction between the surface of the deposited nanoparticles and the substance are based on hydrophobic interactions, physical or chemical adsorption processes (chemisorption), and electrostatic attractions [64, 65]. There is no doubt that without the localized surface plasmon resonance mechanism of the nanoparticles, there would be no sign of enhancement of the surrounding molecules [66]. This observation confirms that SEIRA spectra incorporate significant data on the adsorbed particle and its surrounding molecules [67]. Figure 5 shows the SEIRA spectrum which is considered the enhanced version of the spectrum of BIS and its metabolite without nanoparticles.

Furthermore, the LSPR that resulted from Cit-AgNPs was not limited to SEIRA peaks amplification only, but it also caused limited band shifts in the presence of Cit-AgNPs compared to infrared measurements without nanoparticles as in Fig. 5. The SEIRA band shifts may be a result of the electrostatic and chemisorption interactions between Cit-AgNPs and the active functional groups of BIS and its metabolite such as (-COOH, -C = O, -N, and -OH). These groups have strong interactions with the surface of nanoparticles’ islands as reported in the literature [34]. These surface interactions allowed for stabilizations of BIS and its metabolite to be adsorbed on the surface of the Cit-AgNPs.

### PLSR modeling for SEIRA analysis

Based on the above experimental findings, it was found that the LSPR enhancement produced by Cit-AuNPs is higher than that produced using Cit-AgNPs as shown above. In addition, the time required for the fabrication of the Cit-AuNPs coated glass substrate (1 h) is less than that required to fabricate the Cit-AgNPs coated glass substrate (4 h). As a result, we have selected nanoparticle glass slides coated with Cit-AuNP as a substrate for the quantitative analysis of BIS and the active metabolite in the samples of spiked human plasma.

Chemometrics is a well-established analytical technique and partial least squares regression (PLSR) [25, 26] can make quantitative predictions even in the presence of interfering compounds using the whole SEIRA spectrum without ignoring any part of the spectrum that may include important information. Several methods have applied PLSR chemometric calculation for the analysis of pharmaceutical components [21, 27, 28]. The PLSR models were successfully built using the prepared 25 mixtures of the two analytes, BIS, and the active metabolite. Linear calibration curves were achieved with concentration ranges of (15–240 and 15–240 ng/mL) for BIS and its active metabolite, respectively as shown in Fig. 5S. The concentration range has been selected to cover the reported C_max_ of BIS and its active metabolite in human blood [68].

The pre-processing step is essential to obtain the correct SEIRA signal information of BIS and its active metabolite using the built model [69, 70]. To identify a representative band for BIS and its active metabolite, the spectrum of each compound was measured and plotted as an overlay plotting chart, and then it was matched to the lab-prepared mixtures spectra aiming for identification of the characteristic peaks for each compound Fig. 5C. The whole SEIRA range was recorded between 4600 − 400 cm^− 1^ bands, and the area between (4600 to 4000 cm^− 1^) and (2200 to 1950 cm^− 1^) was identified to contain irregular bands which may be due to carbon dioxide and humidity interference, so we have discarded these regions as a pre-processing treatment before any PLSR modeling. The remaining range/areas between (4000 to 2200 cm^− 1^) and (1950 to 400 cm^− 1^) were used for PLSR modeling. Then, Savitzky-Golay’s first derivative algorithm was applied to the spectra to resolve the overlap between BIS and its active metabolite bands, as illustrated in Fig. 6. Thus, after the pre-processing steps, each recorded SEIRA spectrum gave 1411 data points that were used for the PLSR model using the Unscrambler X® 10.4 software.

Several trials have been performed for the determination of the optimal number of latent variables (LVs) for optimization of the PLSR model [71–73]. The number of LVs selected depends on the analysis of the regression coefficient to ensure that PLSR modeling is based on the characteristic SEIRA bands rather than pseudo-artifacts. Twenty-five calibration mixture spectra were recorded, and the number of LVs was determined by removing one sample at a time and then using the 24 mixtures to determine the concentration of the omitted sample then the process was repeated about 24 times [74]. The concentrations of the omitted samples were then compared to the actual calibration concentrations to get the root mean squared error of cross-validation (RMSECV). The optimal number of LVs for BIS and its active metabolite was two. The predictive ability of the PLSR model can be determined using RMSECV [72].

The plotting of actual concentrations of the calibration set versus the concentration of the predicted samples was utilized to calculate the concentrations of BIS and its active metabolite. Table 2 contains the validation parameters in terms of linearity, range, correlation coefficients (R^2^), standard errors of the calibrations, bias, slope, and intercepts. The calculated values of (R^2^) were close to 1, confirming the good fitting between the predicted and the actual concentrations. A good recovery percentage of about 101.19% was obtained.

Another validation set consisting of 10 mixtures was used to examine the predictive ability of unknown concentrations of the built PLSR model as shown in Table 2 S. The PLSR model that has the optimal number of LVs was used to calculate the predicted concentrations. Good recoveries standard deviations were obtained at 98.82 ± 0.98 and 99.12 ± 1.36 for BIS and its metabolite, respectively indicating method applicability to unknown samples. The small values of RMSEP indicated the high predictability of the PLSR model [75].

**Table 2:** Calibration and validation parameters for the PLSR model

Parameters	Analytes	
	BIS	Active metabolite
***No of samples***	25	25
***Range (ng/mL)***	15–240	15–240
***Slope***	0.9859	0.9906
***Offset (Intercept)***	1.2519	0.9542
***Correlation***	0.9929	0.9953
***R*** ^***2***^ ***(Pearson)***	0.9859	0.9906
RMSEC*	9.2850	8.5684
SEC**	9.4937	8.7609
***Bias***	0	0

* Calibration Root Mean Square Error

** Calibration Square Error

### Bisacodyl and the metabolite analysis in spiked human plasma

To examine the quantitative power of the developed SEIRA-PLSR method for the quantification of BIS and its metabolite in spiked human plasma samples, the prepared Cit-AuNP coated glass substrates were used to extract the concentrations of molecules in spiked human blood plasma. The drugs were screened using the device and the method was applied successfully to quantify BIS and its active metabolite in plasma in concentration ranges similar to the C_max_ reported for BIS [69] as shown in Table 3. These results indicated the power of the SEIRA-PLSR method for the rapid quantitative determinations of drugs in human blood plasma. Both substrates have good performance for quantification of the mixture, whereas AuNPs have more advantages in terms of stability, easier preparation, rapid glass coating, and more accurate results. The only disadvantage of AuNPs is their cost.

**Table 3:** Application of the proposed SERA-PLSR method for simultaneous determination of BIS and its metabolite in spiked human plasma

Analytes	Conc. added (ng/mL)	Conc. found (ng/mL)	Mean Recovery %
**BIS**	26	25.8	99.23
	100	101	101.00
	236	240	101.69
Mean Recovery % ± RSD*	**100.64** ***±*** **1.69**		
**Metabolite**	236	234	99.15
	100	98.7	98.70
	26	25.6	98.46
Mean Recovery % ± RSD*	**98.77** ***±*** **1.41**		

*Average of three determinations

The performance of the current SEIRA-PLSR method was compared to published nanomaterials-based methods [12, 18, 78, 79], and it can be demonstrated that the SEIRA-PLSR has a good performance as illustrated in Table 4. In addition, the performance of the current SEIRA-PLSR was compared to the performance of the already published method [68] for analysis of BIS and its metabolite by analysis of three spiked concentrations (26, 100, and 236 ng/mL) for both BIS and its metabolite using the HPLC-MS/MS described by Fredrich et al. [69]. Similar recoveries were obtained with a relative standard deviation < 2. Moreover, the SEIRA coupling with chemometric PLSR tools permitted the simultaneous quantification of BIS and its metabolite, thus adding a new test for the identification of analytes in human plasma. The proposed SEIRA approach can be extended to the rapid analysis of many other structurally related compounds. Also, it can be used for the detection and analysis of active metabolite in water due to contamination of waste. Future work may involve the development of more user-friendly and cost-effective instrumentation, as well as exploring multimodal approaches by integrating SEIRA with other spectroscopic techniques or imaging modalities. The biomedical and clinical applications of SEIRA could see significant progress, offering potential for early disease diagnosis and monitoring. Additionally, researchers may delve into advancements in materials and nanotechnology, tailoring new nanostructures to enhance the reproducibility and reliability of the technique. It is recommended to stay updated with the latest scientific literature for the most recent developments in SEIRA as the field continues to evolve with ongoing research and technological innovations.

**Table 4:** Nanomaterial-based methods used for analysis of substances in biological fluids in comparison to the current work

Nanomaterial used	Technique	Linearity range	The analyte	Reference
Gold nano-stars and spherical nanoparticles	SEIRS	0.063-2.00 ug/mL 31.25–250.00 ug/mL	Thioglycolic acid Bovine serum albumin	[12]
Silver nanoparticles and TiOx/ZnO nanocomposites	SERS SEIRA	0.25-5.00 mg/L 0.5–25 mg/mL	Crystal violet	[18]
Modified screen-printed electrode on silver nanoparticles	Potentiometry	1.00 × 10^− 6^–1.00 × 10^− 2^ M/L 1.00 × 10^− 8^–1.00 × 10^− 1^ M/L	Lanthanum III	[76]
Prepared gold nanoparticles on a glass substrate	SEIRA	1.00 × 10^− 6^–100 × 10^− 2^ M	Uracil	[17]
Citrate-capped silver nanoparticles Citrate-capped gold nanoparticles	SEIRA	15–240 ng/mL	Bisacodyl	Current work

## Conclusions

We have illustrated the applicability of Cit-AuNPs and Cit-AgNPs for the selective extraction of bisacodyl and its metabolite from human plasma samples and the ability of SEIRA-PLSR detection of low concentration levels. The presence of functional groups such as (-COOH, -C = O, -N, and -OH) facilitated the covalent immobilization of the analytes on a cost-effective Cit-AuNPs coated glass slides as a SEIRA sensor. The formation of a charge-transfer complex and immobilization of the chemisorbed analytes molecules and the nanoparticles hotspots over the glass substrates leads to enhancement in the SEIRA signal of the analytes. The gold nanoparticles showed higher performance in comparison to the silver nanoparticles for the quantitative analysis of BIS and its metabolite in plasma, which may be due to the higher stability, easier preparation, and higher enhancement factor. The demonstrated SEIRA approach for the selective determination of the analytes can be extended to the rapid analysis of many other structurally related compounds in laboratories. Also, it can be used for the detection and analysis of active metabolites in water due to contamination of waste.

### Electronic supplementary material

Below is the link to the electronic supplementary material.

Supplementary Material 1

## Data Availability

All data generated or analyzed during this study are included in this article and the raw data is available from the corresponding author if requested.
