# Supplementary material for: Comparing silver and gold nanoislands’ surface plasmon resonance for bisacodyl and its metabolite quantification in human plasma
Source: BMC Chem. 2024 Mar 23;18(1):56. doi: 10.1186/s13065-024-01157-8 (PMC10960993; doi:10.1186/s13065-024-01157-8)
Supplement: Supplementary file 1 — Supplementary Material 1 [file 13065_2024_1157_MOESM1_ESM.docx]

**Comparing silver and gold nanoislands' surface plasmon resonance for Bisacodyl and its metabolite quantification in human plasma**

**Supplementary figures**


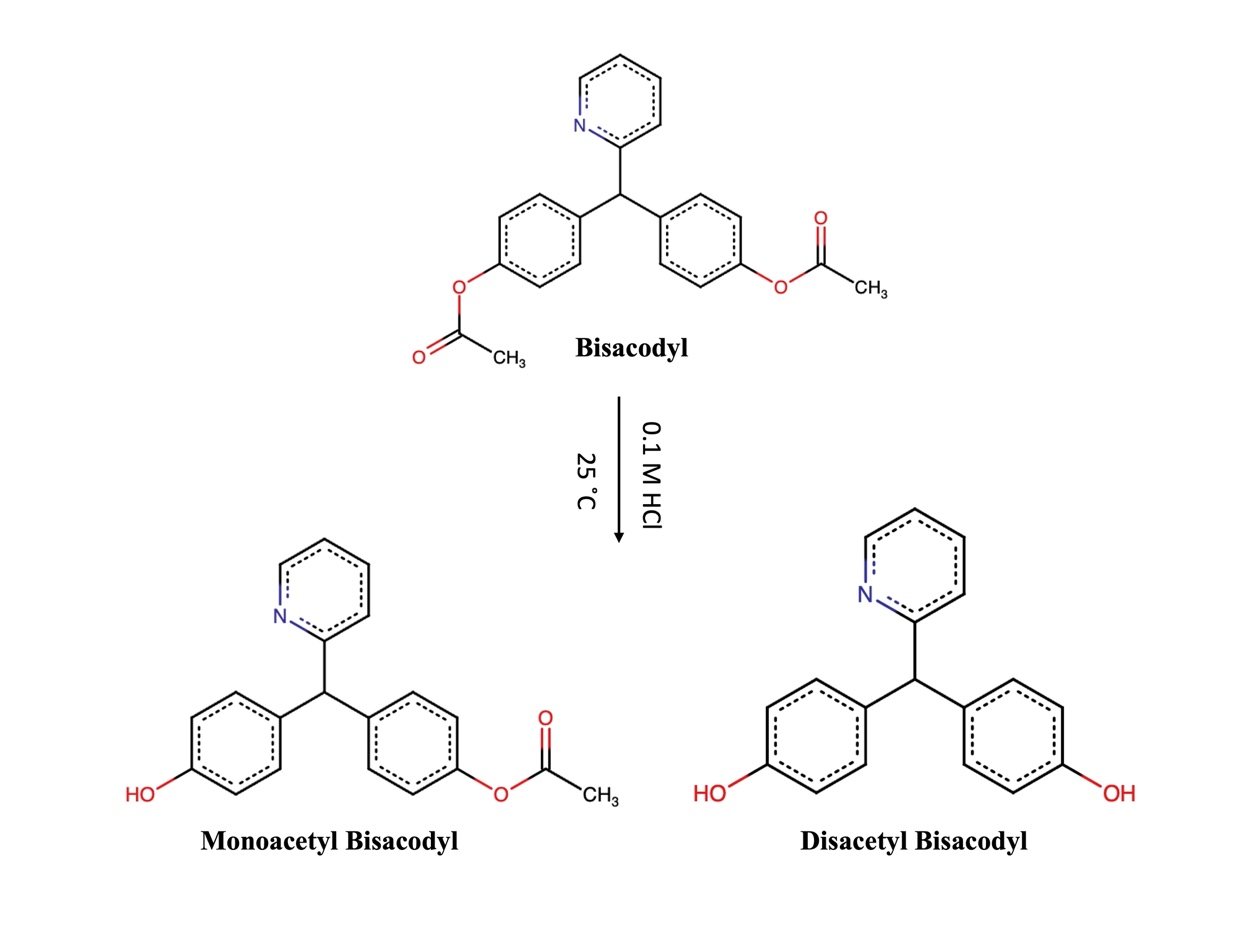


**Figure 1S:** Schematic representation of the degradation pathway of Bisacodyl.

| 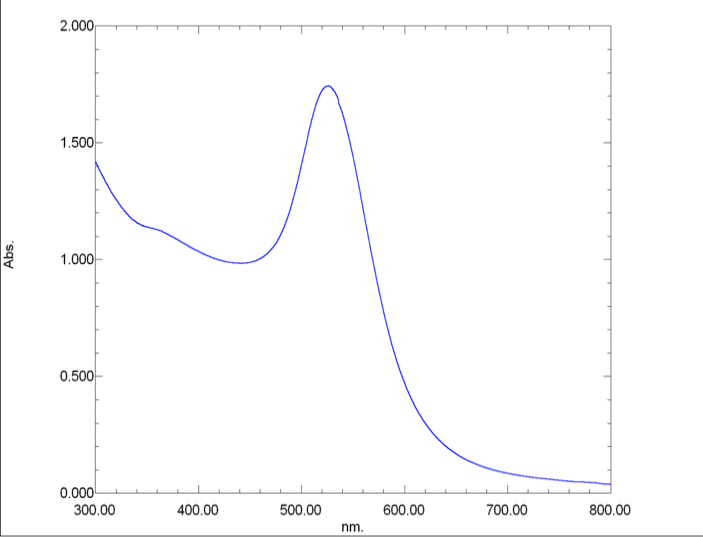a) | b)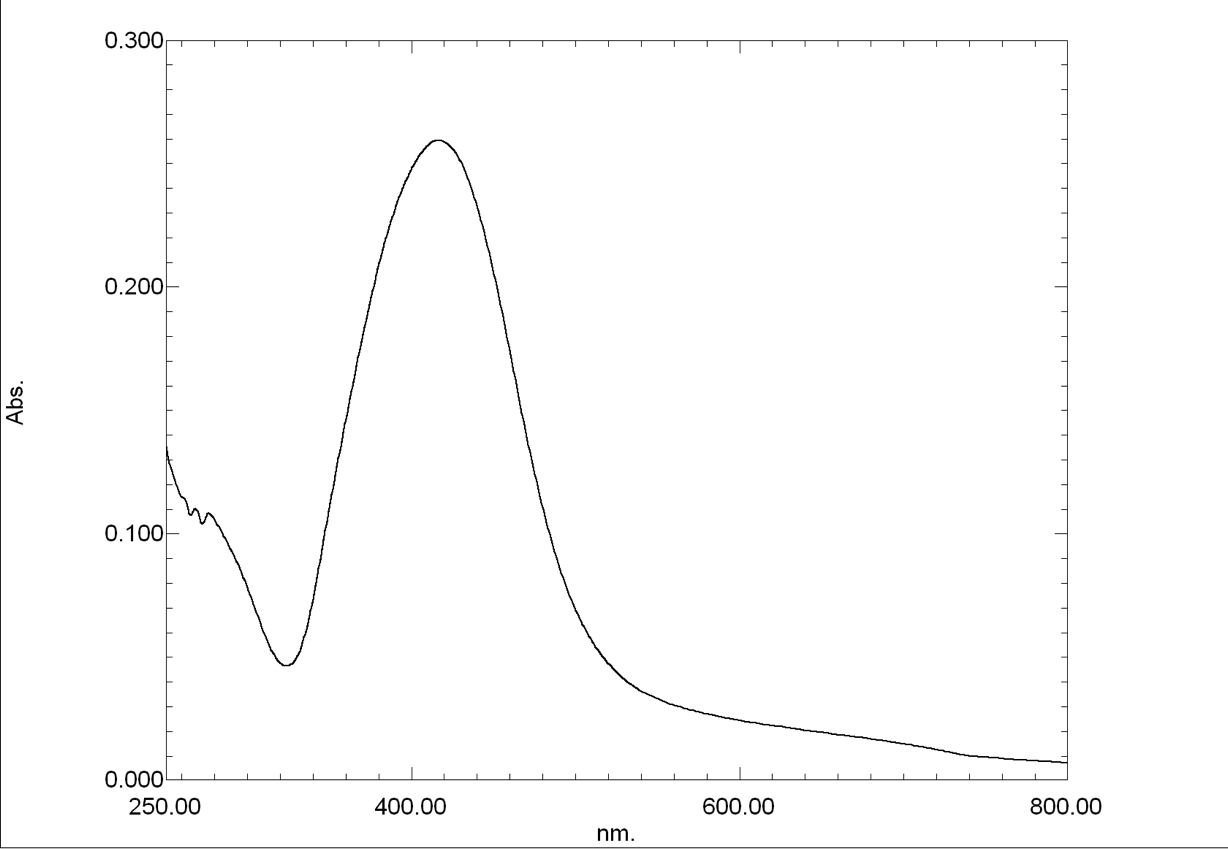 |
| --- | --- |

**Figure 2S:** The UV/VIS spectra of **(a)** Cit-AuNPs showing ג_max_ at 525 nm and **(b)** Cit-AgNPs showing λ_max_ at 415 nm.


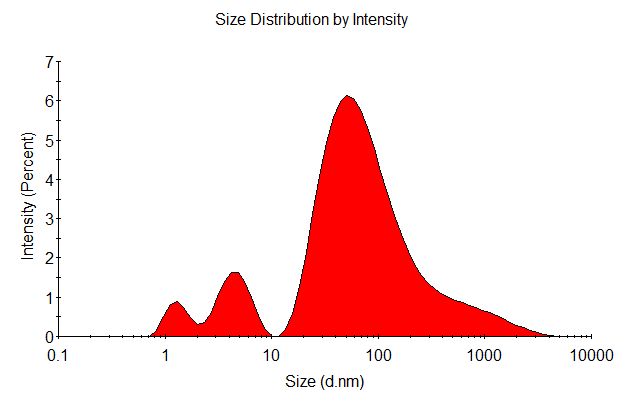

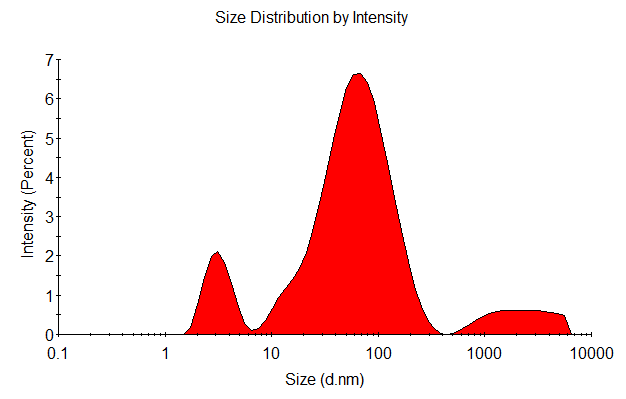


(B)

(A)

**Figure 3S:** Zeta Sizer images showing the average hydrodynamic radius for both (A) Cit-AuNPs and (B) Cit-AgNPs


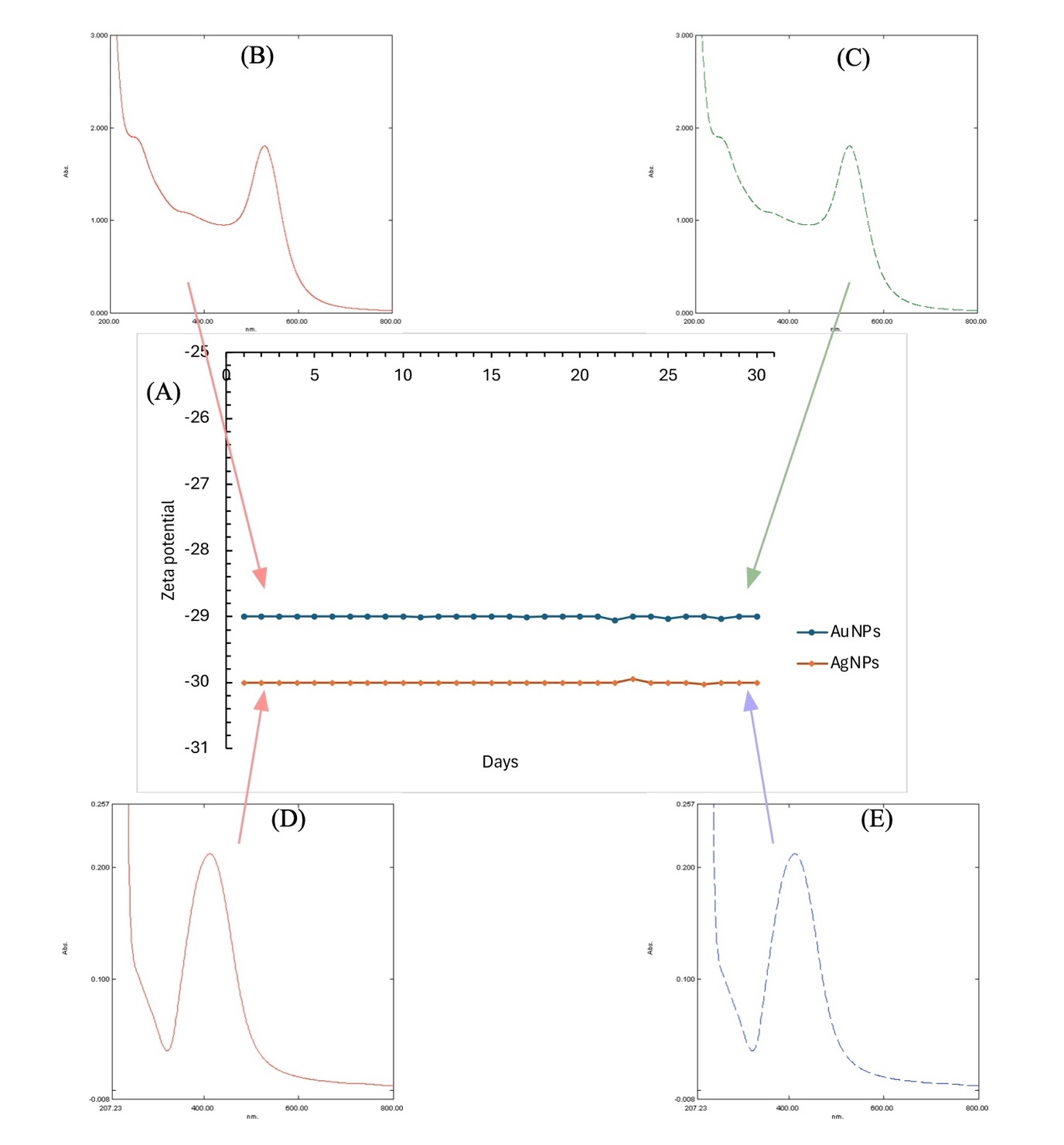


**Figure (4S):** shows the results of the stability experiments, Figure (4S-A) Plot of Zeta potential measured during 30 days for Cit-AuNPs and Cit-AgNPs indicating a negatively charged nanoparticles for the whole duration of the 30 days allowing electrostatic repulsion preventing agglomeration. Figure (4S-B & C) shows UV/Vis spectrum of Cit-AuNPs measured at the beginning and the end of the 30 days indicating that the peak shape, width, and λ_max_ remained unchanged during the measurement period. Figure (4S-C & D) shows UV/Vis spectrum of Cit-AgNPs measured at the beginning and the end of the 30 days indicating that the peak shape, width, and λ_max_ remained unchanged during the measurement period.

**Figure 5S:** Correlation plots between the actual refence concentration values against predicted concentrations for calibration concentrations of (A) Bisacodyl and (B) its metabolite.

**Supplementary tables**

**Table 1S**: Experimental design showed the coded and concentration levels of the 25 mixtures used for calibration following multilevel fractional factorial design.

| ***Coded Levels ^a^*** | |  |  | ***Concentrations (ng/mL) ^b^*** | |
| --- | --- | --- | --- | --- | --- |
| ***BIS*** | ***Active***  ***metabolite*** |  | ***Mixture***  ***Numbers*** | ***BIS*** | ***Active metabolite*** |
| 0 | 0 |  | 1 | 60 | 60 |
| 0 | -2 |  | 2 | 60 | 15 |
| -2 | -2 |  | 3 | 15 | 15 |
| -2 | 2 |  | 4 | 15 | 240 |
| 2 | -1 |  | 5 | 240 | 30 |
| -1 | 2 |  | 6 | 30 | 240 |
| 2 | 0 |  | 7 | 240 | 60 |
| 0 | -1 |  | 8 | 60 | 30 |
| -1 | -1 |  | 9 | 30 | 30 |
| -1 | 1 |  | 10 | 30 | 120 |
| 1 | 2 |  | 11 | 120 | 240 |
| 2 | 1 |  | 12 | 240 | 240 |
| 1 | 0 |  | 13 | 120 | 60 |
| 0 | 2 |  | 14 | 60 | 240 |
| 2 | 2 |  | 15 | 240 | 240 |
| 2 | -2 |  | 16 | 240 | 15 |
| -2 | 1 |  | 17 | 15 | 120 |
| 1 | -2 |  | 18 | 120 | 15 |
| -2 | 0 |  | 19 | 15 | 60 |
| 0 | 1 |  | 20 | 60 | 120 |
| 1 | 1 |  | 21 | 120 | 120 |
| 1 | -1 |  | 22 | 120 | 30 |
| -1 | -2 |  | 23 | 30 | 15 |
| -2 | -1 |  | 24 | 15 | 30 |
| -1 | 0 |  | 25 | 30 | 60 |

**^a^** The coded levels represent 5 concentration levels for each of the two compounds that coded as: (1, -1, 0, -2, 2). Each code represents one of BIS concentrations as (1, -1, 0, -2, 2) corresponds to (120, 30, 60, 15, 240 ng/mL), respectively. Also, each code represents one of BIS active metabolite concentrations as (1, -1, 0, -2, 2) corresponds to (120, 30, 60, 15, 240 ng/mL), respectively.

^b^ The concentration range have been selected to cover the reported C_max_ of BIS and its active metabolite in human blood (Ref. 70).

**Table 2S**: Validation set design showing the coded levels, concentrations of the 10 mixtures and the obtained recoveries.

| ***Coded Levels ^a^*** | | ***Concentrations (ng/mL)*** | | ***Recovery %*** | |
| --- | --- | --- | --- | --- | --- |
| ***BIS*** | ***Active metabolite*** | ***BIS*** | ***Active metabolite*** | ***BIS*** | ***Active metabolite*** |
| 0 | -2 | 60 | 15 | 98.76 | 97.66 |
| -2 | 2 | 15 | 240 | 97.95 | 99.33 |
| -1 | 2 | 30 | 240 | 98.66 | 100.2 |
| 0 | -1 | 60 | 30 | 99.23 | 98.37 |
| -1 | 1 | 30 | 120 | 99.51 | 100.5 |
| 2 | 1 | 240 | 240 | 98.33 | 99.89 |
| 0 | 2 | 60 | 240 | 98.59 | 98.49 |
| 2 | -2 | 240 | 15 | 100.3 | 98.53 |
| 1 | -2 | 120 | 15 | 97.85 | 98.91 |
| 0 | 1 | 60 | 120 | 98.96 | 99.29 |
| *Mean recovery % ± Standard deviation* | | | | 98.82 ± 0.98 | 99.12 ± 1.36 |

^a^ Coded levels selected randomly within the mixtures of the experimental set.
